# Serum Levels of Monocyte Chemoattractant Protein-1 and All-Cause and Cardiovascular Mortality among Patients with Coronary Artery Disease

**DOI:** 10.1371/journal.pone.0120633

**Published:** 2015-03-18

**Authors:** Ding Ding, Dongfang Su, Xinrui Li, Zhongxia Li, Yujie Wang, Jian Qiu, Puqing Lin, Yuan Zhang, Pi Guo, Min Xia, Dan Li, Yan Yang, Gang Hu, Wenhua Ling

**Affiliations:** 1 Guangdong Provincial Key Laboratory of Food, Nutrition and Health, Department of Nutrition, School of Public Health, Sun Yat-sen University, Guangzhou, Guangdong, China; 2 Chronic Disease Epidemiology Laboratory, Pennington Biomedical Research Center, Baton Rouge, Louisiana, United States of America; 3 Department of Cardiology, General Hospital of Guangzhou Military Command of People’s Liberation Army, Guangdong, China; 4 Department of Medical Statistics and Epidemiology, School of Public Health, Sun Yat-sen University, Guangzhou, Guangdong, China; German Diabetes Center, Leibniz Center for Diabetes Research at Heinrich Heine University Duesseldorf, GERMANY

## Abstract

**Background:**

Monocyte chemoattractant protein-1 (MCP-1) is an important chemokine at multiple phases of atherosclerosis in animals, but human studies are few and inconsistent. The aim of this study is to investigate the association of serum MCP-1with all-cause and cardiovascular disease (CVD) mortality among coronary artery disease (CAD) patients and determine whether this biomarker can add secondary prognostic value to standard risk predictors.

**Methods:**

MCP-1 was measured at baseline in 1411 CAD patients who were 40–85 years of age. Cox proportional hazards regression models were used to estimate the association of MCP-1 levels with death risk.

**Results:**

During a median follow-up of 3.3 years, 117 deaths were recorded, 88 of which were due to CVD. The multivariable-adjusted hazard ratios across tertiles of MCP-1 were 1.51 (95% confidence intervals [CI] 0.89–2.58), 1.00, and 2.11 (95% CI 1.31–3.40) for all-cause mortality, and 1.50 (95% CI 0.80–2.81), 1.00, and 2.21 (95% CI 1.27–3.87) for CVD mortality. The addition of serum MCP-1 to the fully adjusted model increased the C-index by 0.009 (p<0.0001) for all-cause mortality and 0.008 (p<0.0001) for CVD mortality and significantly improved the predictive ability by 12.1% (P = 0.006) on all-cause mortality and 12.6% (P = 0.003) on CVD mortality using the net reclassification improvement method.

**Conclusions:**

Both lower and higher MCP-1 levels are associated with an increased risk of all-cause and CVD mortality among CAD patients. More research is needed to confirm its clinical relevance.

## Introduction

Cardiovascular disease (CVD) is the leading cause of death and disability in China and worldwide, and it is projected by the World Health Organization to be the greatest cause of death in the world within the next 15 years [1]. Since coronary artery disease (CAD) patients are at high risk of recurrent cardiovascular events and premature deaths, more attention is needed on the risk prediction and secondary prevention of CAD patients.

It is widely accepted that inflammation plays a crucial role in both initiation and progression of CAD, and a lot of immune cells and chemokines are involved in the inflammatory pathway. C-reactive protein (CRP) and IL-6 have been widely studied as effective biomarkers and independent risk factors for CAD [2]. Although CRP is a reliable marker of inflammation, several other inflammatory mediators are critically involved in the pathogenesis of CAD and may serve as potential biomarkers providing additional prognostic information, i.e. monocyte chemoattractant protein-1 (MCP-1) [3, 4]. MCP-1 is a chemokine responsible for the recruitment of monocytes to sites of inflammation, while monocytes play a key role in various phrases of CAD, including initiation of the fatty streak, promotion of plaque instability, as well as remodeling and restenosis after myocardial infarction [5, 6]. Animal experiments have found that the expression level of MCP-1 is directly associated with the extent of atherosclerosis and the infiltration of monocytes into the atherosclerotic lesion [7, 8]. However, population studies on the association of MCP-1 with CVD or death risk among CAD patients are still limited. Several small case-control studies find MCP-1 level is higher among CAD patients than in healthy subjects [9, 10]. Only 4 cohort studies have analyzed the association between MCP-1 levels and death risk, and 3 of them recruited acute coronary syndrome (ACS) patients, and the other one collected patients with ischemic chest pain [3, 4, 11, 12]. None of them recruited stable CAD patients at baseline. Thus, the aim of the present study is to evaluate whether MCP-1 can add prognostic value to traditional risk factors for Chinese patients with ACS and stable CAD.

## Methods

### Participants

The recruitment of the Guangdong Coronary Artery Disease Cohort was between October 2008 and December 2011 [13, 14]. We enrolled 1980 successive patients admitted to the Cardiology Department of 3 superior specialty hospitals in Guangdong and diagnosed as CAD [International Classification of Diseases (ICD)-10 codes I20-I25] according to World Health Organization 1999/2000 guidelines [15, 16]. Eligible diseases were defined as the occurrence of at least two of the following situations: 1) typical chest pain with the property of oppression, dullness and constriction which occurred in the middle and upper part of sternum before, 2) ST-segment deviation of 0.05 mV or more and/or T wave inversion on first or histological electrocardiograph, 3) increased troponin and/or creatine kinase MB on initial blood tests, and/or at least one of the following situations: 1) measurable stenotic valve lesions of coronary arteries by coronary angiography, and 2) history of percutaneous coronary intervention or coronary artery bypass grafting. Generally, when patients entered the Department of Cardiology, eligibility was reviewed by one pathologist and two cardiologists in the specific hospital. After excluding 569 participants because of missing MCP-1 measurements, the final sample comprised 1411 CAD patients aged 40 to 85 years (S1 Fig.). S1 Table showed the comparison between retained and excluded participants. Compared with the retained participants, those excluded from the present analysis were generally younger (62.4 vs. 64.1 years). There were no significant differences of other relative baseline characteristics between included and excluded participants. The study was approved by the Sun Yat-sen University ethics committee, and all participants signed the informed consent.

### Clinical measurements

A standardized questionnaire on general information of examination date, birth date, gender, education level, leisure-time physical activity, smoking habits, alcohol consumption, family history of CAD, medication history, and a validated food frequency questionnaire [17] were conducted through a face-to-face interview. Smoking was defined as at least one cigarette a day and lasting more than six months. Alcohol drinking was defined as drinking any type of alcoholic beverage at least once a week and lasting more than six months. Smoking and drinking status was classified as never, past, or current.

Clinical characteristics, clinical tests’ results and treatment of participants were collected from an electronic case record system. At admission, trained nurses measured height, weight and blood pressure using a standard protocol. BMI was calculated by dividing weight in kilograms by the square of height in meters. Glomerular filtration rate (GFR) was used to assess renal function according to the most recent Modification of Diet in Renal Disease Study equation [18], which is estimated at GFR = 175 × (standardized serum creatinine in mg/dL) ^−1.154^ × Age^−0.203^ × 0.742 (if female). Treatment information of CAD included percutaneous coronary intervention and coronary artery bypass graft. Venous blood samples were drawn in the next morning after hospital admission with at least 12 hours fasting. Lipids and fasting plasma glucose (FPG) were determined by standard methods immediately after collection. Blood samples were stored at −80° C until thawed and then analyzed. Serum levels of MCP-1 and CRP were measured with a FlowCytomix technique using FlowCytomix Human Basic Kit (BMS8420FF, eBioscience, USA) together with Human MCP-1 FlowCytomix Simplex Kit (BMS8281FF, eBioscience, USA) and Human CRP FlowCytomix Simplex Kit (BMS82288FF, eBioscience, USA) on a BD FACSCalibur instrument (BD Biosciences, USA). Data were obtained from CellQuest software (BD Biosciences, USA) and calculated by the FlowCytomix Program (eBioscience, USA). The limit of detection was 18.2pg/mL for MCP-1 and 0.1mg/L for CRP. The mean intra-assay and inter-assay coefficients of variation were 10%, 13% for MCP-1and 9%, 14% for CRP.

### Prospective follow-up

Follow-up data were collected from hospitals’ medical records of readmission, telephone contacts with patients or family members, and death registration of the Guangdong Provincial Center for Disease Control and Prevention which provided detailed causes of death. The surveys were followed to the end of July 2013 or patients’ death, whichever occurred first. The ICD codes were used to code the cause of death, and the ICD codes I00-I99 were classified as CVD deaths.

### Statistical analysis

Serum MCP-1 levels were log-transformed and then classified into tertiles(<33.3%, 33.3 to <66.7% [reference group], and ≥66.7%). Differences in normally distributed continuous variables among three groups were analyzed by the general linear model after adjustment for age. A chi-square test was used for categorical variables and a Kruskal-Wallis one-way ANOVA was used for continuous variables that were not normally distributed. The associations between baseline serum MCP-1 levels and the risks of all-cause and CVD mortality were analyzed by using Cox proportional hazards models. All analyses were adjusted for age and gender, and further for education, leisure-time physical activity, smoking, alcohol drinking, BMI, systolic blood pressure, FPG, low-density lipoprotein cholesterol, GFR, history of heart failure, and use of antihypertensive, anti-diabetic, cholesterol-lowering, and anti-platelet drugs. CRP (log transformed) was included in the final models. We used restricted cubic splines in Cox models to test whether there was a dose-response or non-linear association of MCP-1 as a continuous variable with all-cause and CVD mortality risk. The C-index associated with the risk-estimation model was calculated based on all the classic risk factors listed above, and the likelihood ratio test was used to compare the discrimination of the models including and excluding MCP-1 [19, 20]. Net reclassification improvement (NRI) was further used to assess the contribution of MCP-1 [21]. We stratified patients into four risk categories (<5%, 5 to <10%, 10 to <15%, and ≥15%) based on the clinical variables. Statistical significance was considered to be P<0.05. All statistical analyses were performed with IBM SPSS Statistics 20.0 (IBM SPSS Inc, Chicago, III), SAS for Windows, version 9.3 (SAS Institute, Cary, NC), and R for Windows, version 3.0.1.

## Results

At baseline, only age, FPG, CRP, CAD types, GFR, history of heart failure, and treatment with percutaneous coronary intervention were different among MCP-1 tertiles (Table 1).

**Table 1:** Baseline characteristics according to serum MCP-1 level among coronary artery disease patients.

	Baseline serum MCP-1 level			P for difference
	<33.3%	33.3 to <66.7%	≥66.7	
MCP-1 (pg/mL)	<200.4 (male)<231.1 (female)	200.4–354.5 (male)231.1–360.6 (female)	>354.5 (male)>360.6 (female)	
No. of participants (%)	470	468	473	
Male (%)	65.3	65.4	65.1	1.00
Age at baseline (yrs)	62.7 (0.5)	64.6 (0.5)	65.1 (0.5)	0.002
Body mass index (kg/m^2^)	23.9 (0.2)	23.9 (0.2)	23.9 (0.2)	0.97
Fasting plasma glucose (mmol/L)	6.70 (0.12)	6.36 (0.12)	6.32 (0.12)	0.04
Systolic blood pressure (mm Hg)	135 (1.0)	134 (1.0)	134 (1.0)	0.84
Diastolic blood pressure (mm Hg)	77 (0.6)	77 (0.6)	76 (0.6)	0.27
Low-density lipoprotein cholesterol (mmol/L)	3.00 (0.05)	2.93 (0.05)	3.01 (0.05)	0.32
High-density lipoprotein cholesterol (mmol/L)	1.10 (0.01)	1.07 (0.01)	1.06 (0.01)	0.06
C-reactive protein (mg/L)	5.72 (1.24–17.3)	2.55 (0.75–10.2)	3.33 (1.15–10.6)	<0.001
Duration of CAD (yrs)				
First diagnosed CAD (n = 767)	-	-	-	
History of CAD (n = 644)	2.91 (0.87–8.03)	2.53 (0.70–7.50)	2.96 (0.75–8.00)	0.83
Married (%)	92.0	90.7	91.3	0.85
Years of education (%)				0.67
≤9	62.3	62.3	61.7	
10–12	22.1	18.7	19.6	
≥13	15.6	19.0	18.6	
Smoking (%)				0.63
Never	60.2	61.5	62.7	
Past	8.8	10.9	9.0	
Current	31.0	27.6	28.4	
Alcohol drinking (%)				0.80
Never	77.3	77.1	80.5	
Past	7.6	7.8	6.2	
Current	15.0	15.1	13.3	
Leisure-time physical activity (%)				0.70
None	35.6	32.0	32.7	
<30 minutes/day	22.7	21.6	20.8	
≥30 minutes/day	41.7	46.3	46.5	
Type of CAD (%)				<0.001
Acute coronary syndrome	68.5	53.2	55.2	
Chronic CAD	31.5	46.8	44.8	
No. of diseased vessels in coronary angiography (n = 914)				0.32
0	6.8	6.0	8.3	
One-vessel disease	24.4	22.4	17.4	
Two-vessel disease	23.4	23.7	20.2	
Three-vessel disease	45.5	47.8	54.1	
Glomerular filtration rate (mL/min/1.73m^2^), (%)				<0.001
≥90	36.5	27.1	24.2	
60–89	45.1	49.1	48.9	
30–59	17.4	22.1	22.4	
15–29	0.9	1.1	3.1	
<15	0.2	0.7	1.3	
History of diseases (%)				
Hypertension	61.1	61.5	59.0	0.70
Diabetes	23.0	20.1	27.1	0.04
Dyslipidemia	29.1	31.4	31.1	0.72
Heart failure	48.7	37.8	41.9	0.003
Use of medication before admission (%)				
Anti-diabetic drugs	15.0	15.0	19.6	0.10
Antihypertensive drugs	48.1	52.8	52.3	0.28
ACE inhibitors	20.7	14.2	15.5	0.02
Angiotensin II antagonists	18.6	22.7	21.0	0.29
Calcium antagonists	25.2	24.7	24.2	0.93
β-blockers	29.1	30.0	29.9	0.94
Diuretics	7.5	7.9	8.9	0.72
Lipid-lowering drugs	11.8	14.6	14.6	0.34
Anti-platelet drugs	18.4	24.9	25.0	0.02
Treatment of CAD (%)				
Coronary artery bypass graft	3.0	2.1	2.5	0.72
Percutaneous coronary intervention	60.6	51.9	53.1	0.01

Continuous data are reported as mean (standard error) if normally distributed and median (25th, 75th percentile) if non-normally distributed, categorical data are reported as percentages. All normally distributed continuous variables are adjusted for age.

MCP-1, monocyte chemoattractant protein-1; CAD, coronary artery disease.

During a median follow-up of 3.3 years, 117 deaths were recorded, 88 of which were due to CVD. The 29 non-CVD deaths comprised 14 deaths of cancer, 10 deaths of asphyxia due to lung disease, and 5 deaths of other reasons. Since the interactions between gender and MCP-1 levels on the risk of all-cause and CVD mortality were not statistically significant, data for males and females were combined in the analyses to maximize the statistical power. After adjustment for major conventional CVD risk factors, serum MCP-1 showed non-linear associations with all-cause and CVD mortality (Table 2). The multivariable-adjusted hazard ratios (HRs) across three MCP-1 categories were 1.61 (95% confidence intervals [CI] 0.95–2.72), 1.00, and 2.12 (95% CI 1.31–3.42) for all-cause mortality (*P*_trend_ = 0.009), and 1.56 (95% CI 0.84–2.92), 1.00, and 2.23 (95% CI 1.27–3.89) for CVD mortality (*P*_trend_ = 0.02). After further adjustment for serum CRP levels, MCP-1 remained related to risks of all-cause and CVD mortality with this non-linear association. The addition of serum MCP-1 to the fully adjusted model increased the C-index from 0.811 to 0.820 (*P*<0.0001) for all-cause mortality, and 0.827 to 0.835 (*P*<0.0001) for CVD mortality. When we added MCP-1 to the clinical variables to predict all-cause and CVD mortality risk, 17.9% and 14.8% of dead patients were correctly reclassified to higher risk category and 6.0% and 2.3% incorrectly reclassified to lower risk category. Similarly, 2.3% and 2.6% of survive patients were correctly reclassified to lower risk category and 2.1% and 2.5% incorrectly reclassified to higher risk category. So the estimated NRI was 12.1% (95% CI 3.5–20.9%, P = 0.006) for all-cause mortality and 12.6% (95% CI 4.3–20.9%, P = 0.003) for CVD mortality by including MCP-1 (Table 3).

**Table 2:** Hazard ratios for all-cause and cardiovascular mortality according to serum MCP-1levels.

	Baseline serum MCP-1 level			*P*_trend_
	<33.3%	33.3 to <66.7%	≥66.7	
No. of subjects	470	468	473	
Person-years	1477	1586	1602	
All-cause mortality				
No. of death	33	27	57	
Adjustment for age and gender	1.42 (0.86–2.37)	1.00	1.98 (1.25–3.14)	0.01
Multivariable adjustment*				
Model 1*	1.46 (0.87–2.43)	1.00	2.01 (1.27–3.19)	0.01
Model 2^†^	1.61 (0.95–2.72)	1.00	2.12 (1.31–3.42)	0.009
Model 3^‡^	1.51 (0.89–2.58)	1.00	2.11 (1.31–3.40)	0.01
Cardiovascular mortality				
No. of death	24	19	45	
Adjustment for age and gender	1.46 (0.80–2.66)	1.00	2.23 (1.30–3.81)	0.01
Multivariable adjustment*				
Model 1*	1.52 (0.83–2.79)	1.00	2.27 (1.32–3.90)	0.01
Model 2^†^	1.56 (0.84–2.92)	1.00	2.23 (1.27–3.89)	0.02
Model 3^‡^	1.50 (0.80–2.81)	1.00	2.21 (1.27–3.87)	0.02

*Model 1 was adjusted for age, gender, education, leisure-time physical activity, smoking, and alcohol drinking.

^†^Model 2 was adjusted for model 1 covariates plus history of heart failure, body mass index, systolic blood pressure, fasting plasma glucose, low-density lipoprotein cholesterol, glomerular filtration rate, use of antihypertensive, anti-diabetic, cholesterol-lowering, and anti-platelet drugs.

^‡^ Model 3 was adjusted for model 2 covariates plus C-reactive protein.

MCP-1, monocyte chemoattractant protein-1.

**Table 3:** Reclassification of predicted risk with the addition of MCP-1 in coronary artery disease patients.

Predicted risk (without MCP-1)	Reclassified predicted risk (with MCP-1)				% (N) of subjects reclassified		
	<5%	5 to <10%	10 to <15%	≥15%	Increased risk	Decreased risk	Net correctly reclassified (%)
All-cause mortality							
Dead patients (117)							
<5%	40	8	0	0	17.9	6.0	11.9
5 to <10%	4	21	9	2	(21)	(7)	
10 to <15%	0	0	9	2			
≥15%	0	0	3	19			
Survival patients(1294)							
<5%	1134	13	1	0	2.1	2.3	0.2
5 to <10%	13	70	8	0	(27)	(30)	
10 to <15%	0	11	16	5			
≥15%	0	0	6	17			
NRI (95% CI)							12.1 (3.5–20.9)
							P = 0.006
Cardiovascular mortality							
Dead patients (88)							
<5%	36	5	0	0	14.8	2.3	12.5
5 to <10%	0	19	4	1	(13)	(2)	
10 to <15%	0	1	5	3			
≥15%	0	0	1	13			
Survival patients(1323)							
<5%	1159	19	0	0	2.5	2.6	0.1
5 to <10%	19	68	10	0	(33)	(34)	
10 to <15%	0	10	11	4			
≥15%	0	0	5	18			
NRI (95% CI)							12.6 (4.3–20.9)
							P = 0.003

MCP-1, monocyte chemoattractant protein-1; NRI, net reclassification improvement.

We did additional analyses on subpopulations stratified by BMI or CRP, the non-linear association of MCP-1 with the risk of all-cause and CVD mortality was only present in patients with BMI below 24 kg/m^2^ or CRP above 3.0 mg/L (data not shown). We further checked the interactions between CAD subtypes and MCP-1 levels on the risks of all-cause and CVD mortality. Although the interactions were statistically significant, we didn’t stratify the data by CAD subtypes due to the small number of events in the subgroups.

## Discussion

In this large and hospital-based population of Chinese CAD patients, we found a non-linear association between MCP-1 levels and the risks of all-cause and CVD mortality, independent of conventional CVD risk factors and CRP. CAD patients with higher MCP-1 levels were significantly associated with increased risks for all-cause and CVD mortality.

Previous research found that MCP-1 levels were associated with several traditional risk factors of CAD and subclinical atherosclerosis [4, 22]. Several case-control studies with small sample sizes (from 50 to 76 subjects) also found the MCP-1 level was higher in CAD patients than in healthy subjects, especially among patients with ACS [10, 23, 24]. An Italian study of 50 CAD patients who underwent percutaneous transluminal coronary angioplasty (PTCA) found that MCP-1 levels significantly increased among restenotic patients compared with nonrestenotic patients, and the increase was more significant in the samples collected after 15 and 180 days after PTCA compared with that in the samples collected as early as 24 hours after the procedure [24]. This suggested that the induction and high expression of MCP-1 might be sustained to the late phase of acute coronary event compared with CRP which increased markedly in the acute phase only.

Four cohort studies have evaluated the association between baseline MCP-1 levels and death risk among CAD patients. Two posthoc analyses of clinical trials gave inconsistent findings [3, 4]. MCP-1 level was positively associated with the risk of all-cause mortality among ACS patients in the A to Z trial, but there was no significant association between MCP-1 levels and death risk in the Oral Glycoprotein IIb/IIIa Inhibition with Orbofiban in Patients with Unstable Coronary Syndromes (OPUS-TIMI 16) trial. Other two cohort studies with small sample sizes also found a positive association between baseline MCP-1 level and death risk [11, 12]. However, all these four studies only recruited ACS patients and excluded CAD patients in the stable phase. In the A to Z trial, they further measured MCP-1 levels at 4 months after ACS onset to reflex chronic and stable phase of CAD, and still found a positive association between MCP-1 levels and death risk [3]. The result from the A to Z trial suggested that the positive association of MCP-1 levels with death risk was the same among CAD patients at both acute and stable phases. However, we found a non-linear association between MCP-1 levels and death risk among CAD patients, which was different from previous findings. Several differences between our study and above studies limit a direct comparison of all the results. First, study subjects in two trials were treated with particular drugs which had an influence on both MCP-1 levels and the subsequent events; second, the two previous trials had short follow-up time (10–18 months) compared with our study (mean 3.3 years); third, two cohorts had smaller study sample sizes (183 and 216 subjects) and lower death rates, which limit their applicability to find the association.

Previous basic and animal studies have confirmed that MCP-1 plays an important role in the initiation, development, and progression of CAD. Produced mainly by monocytes, smooth muscle cells, and endothelial cells within atherosclerotic plaques, MCP-1recruits monocytes or macrophages and induces them to migrate to the sites of inflammation [8, 25]. Besides the inflammation reaction, MCP-1 also regulates tissue factor expression and interferes the nitric oxide synthesis, proliferates and migrates the smooth muscle cells, neovascularizes the atheromatous plaque and makes it instable, and participates in the oxidative stress reaction [5–7, 26, 27]. Through all these possible mechanism pathways, MCP-1 is involved in the pathogenesis from the early stage of atherosclerosis development to the reperfusion injury and ventricular remodeling after myocardial infarction. However, the non-linear association from the present study reflected that inflammation was not entirely deleterious or entirely beneficial. This can be explained by the finding of Nahrendorf M et al [28]. The monocyte response after myocardial infarction is temporally biphasic, and a well-coordinated biphasic monocyte response is necessary for proper healing. MCP-1 is released during phase 1(days 1 to 4) after myocardial infarction and recruits proinflammatory monocytes to promote digestion of infracted tissue and removal of necrotic debris, which is followed by active resolution of inflammation and tissue repair in phase 2. Thus, insufficient numbers of MCP-1 and proinflammatory monocytes may delay wound healing. However, if inflammatory monocytes persist too long, the reparative functions of monocytes may be impaired. Therefore, there exists an optimum amount of inflammatory monocytes recruitment due to MCP-1to sites of injury for better prognosis.

Although the present study indicates that MCP-1 levels may be valuable for prediction of secondary prognosis on CAD patients, these findings require further confirmation from other populations. Since the association was non-linear, future studies are needed to find out the proper cut point for risk prediction in the clinical practice. Recently, a number of animal intervention studies including lipid-lowering, anti-diabetic drugs, hormone replacement, and red wine have been shown to effectively reduce the MCP-1 levels [29–32]. Thus, more studies are needed to confirm this effectiveness in human clinical trials.

There are several limitations in our study. First, our subjects were enrolled from hospitals which may bring selection bias. In general, in-patients are considered having severer disease status than non-hospitalized people. However, we included both acute CAD patients and those with stable manifestation, and some of them were electively admitted patients with mild status. Thus we can reduce the bias. Besides, since our participants were all CAD patients, this limited the generalizability of our findings. Second, we cannot completely exclude the effects of residual confounding resulting from measurement error in the assessment of confounding factors or some unmeasured factors. Third, the present study was a cohort study based on patients already with CAD. We could not get causal inference from the present data. Fourth, age was significantly different between excluded and retained participants (62.4 vs. 64.1 years), which may bring selection bias.

In conclusion, there was a non-linear association between MCP-1 and death risk among CAD patients. The addition of MCP-1 to the fully multivariable-adjusted models modestly but significantly improved the discrimination for all-cause and CVD mortality. Thus, MCP-1 may be considered as a biomarker for risk prediction for CAD patients and more investigations of MCP-1 as a therapeutic target are needed.

## Supporting Information

S1 Fig Flow chart illustrating the recruitment of the patients for the study. (DOCX)

S2 Fig Spline plots displaying the risk cardiovascular mortality over the range of MCP-1 (log transformed). (TIF)

S1 Table Characteristics of included and excluded participants. (DOCX)
