# Supplementary material for: Serum Levels of Monocyte Chemoattractant Protein-1 and All-Cause and Cardiovascular Mortality among Patients with Coronary Artery Disease
Source: PLoS One. 2015 Mar 18;10(3):e0120633. doi: 10.1371/journal.pone.0120633 (PMC4365005; doi:10.1371/journal.pone.0120633)
Supplement: S1 Fig — (DOCX) [file pone.0120633.s001.docx]

**S1 Fig. Flow chart illustrating the recruitment of the patients for the study.**

**2831 eligible patients**

**2312 signed informed consent**

519 patients excluded:

1) 362 unwilling to enter;

2) 104 missed any essential information;

3) 53 requested withdrawal.

Clinical measurement

Venous blood sample collection

Standardized questionnaire

**1980 annual follow-up visit**

**1411 in final analysis**

569 patients excluded:

Inadequate blood samples for MCP-1 analyses.
