# Supplementary figures and images for: Serum Levels of Monocyte Chemoattractant Protein-1 and All-Cause and Cardiovascular Mortality among Patients with Coronary Artery Disease
Source: PLoS One. 2015 Mar 18;10(3):e0120633. doi: 10.1371/journal.pone.0120633 (PMC4365005; doi:10.1371/journal.pone.0120633)

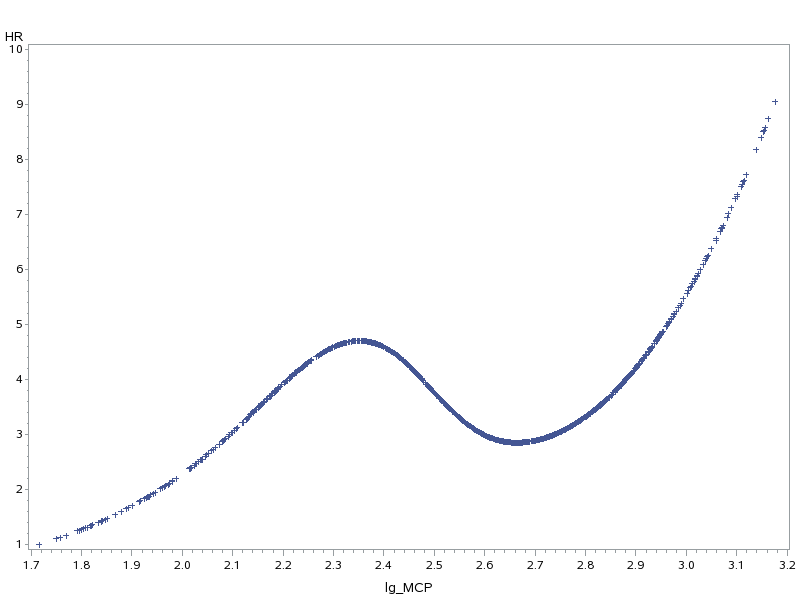

Supplement: S2 Fig — (TIF) [file pone.0120633.s002.tif]
