# Supplementary material for: Serum Levels of Monocyte Chemoattractant Protein-1 and All-Cause and Cardiovascular Mortality among Patients with Coronary Artery Disease
Source: PLoS One. 2015 Mar 18;10(3):e0120633. doi: 10.1371/journal.pone.0120633 (PMC4365005; doi:10.1371/journal.pone.0120633)
Supplement: S1 Table — (DOCX) [file pone.0120633.s003.docx]

**S1 Table. Characteristics of included and excluded participants.**

|  | Included  n=1411 | Excluded  n=569 | P value |
| --- | --- | --- | --- |
| Male (%) | 65.3 | 66.4 | 0.62 |
| Age at baseline (yrs) | 64.1 (0.3) | 62.4 (0.5) | 0.002 |
| Body mass index (kg/m^2^) | 23.9 (0.1) | 23.7 (0.1) | 0.21 |
| Fasting plasma glucose (mmol/L) | 6.46 (0.07) | 6.55 (0.12) | 0.48 |
| High-density lipoprotein cholesterol (mmol/L) | 1.08 (0.01) | 1.10 (0.01) | 0.21 |
| C-reactive protein (mg/L) | 3.69 (0.99 -13.5) | 4.54 (1.15-13.9) | 0.18 |
| Type of CAD (%) |  |  | 0.16 |
| Acute coronary syndrome | 59 | 62.4 |  |
| Chronic CAD | 41 | 37.6 |  |
| Glomerular filtration rate (mL/min/1.73m^2^), (%) |  |  | 0.77 |
| ≥90 | 29.3 | 27.6 |  |
| 60-89 | 47.7 | 49.3 |  |
| 30-59 | 20.6 | 20.3 |  |
| 15-29 | 1.7 | 2.4 |  |
| <15 | 0.7 | 0.5 |  |
| History of diseases (%) |  |  |  |
| Diabetes | 25.1 | 24.8 | 0.89 |
| Heart failure | 42.8 | 41.5 | 0.59 |
